# Extensive Reannotation of the Genome of the Model Streptomycete *Streptomyces lividans* TK24 Based on Transcriptome and Proteome Information

**DOI:** 10.3389/fmicb.2021.604034

**Published:** 2021-04-14

**Authors:** Julian Droste, Christian Rückert, Jörn Kalinowski, Mohamed Belal Hamed, Jozef Anné, Kenneth Simoens, Kristel Bernaerts, Anastassios Economou, Tobias Busche

**Affiliations:** ^1^Microbial Genomics and Biotechnology, Center for Biotechnology, Bielefeld University, Bielefeld, Germany; ^2^Laboratory of Molecular Bacteriology, Department of Microbiology and Immunology, KU Leuven, Rega Institute, Leuven, Belgium; ^3^Molecular Biology Department, National Research Centre, Dokii, Egypt; ^4^Bio- and Chemical Systems Technology, Reactor Engineering, and Safety (CREaS) Section, Department of Chemical Engineering, KU Leuven, Leuven, Belgium

**Keywords:** *Streptomyces lividans* TK24, reannotation, promoter, RNA sequencing (RNA-seq), *cis*-regulatory elements, attenuation, secondary metabolite gene clusters

## Abstract

*Streptomyces lividans* TK24 is a relevant Gram-positive soil inhabiting bacterium and one of the model organisms of the genus *Streptomyces*. It is known for its potential to produce secondary metabolites, antibiotics, and other industrially relevant products. *S. lividans* TK24 is the plasmid-free derivative of *S. lividans* 66 and a close genetic relative of the strain *Streptomyces coelicolor* A3(2). In this study, we used transcriptome and proteome data to improve the annotation of the *S. lividans* TK24 genome. The RNA-seq data of primary 5′-ends of transcripts were used to determine transcription start sites (TSS) in the genome. We identified 5,424 TSS, of which 4,664 were assigned to annotated CDS and ncRNAs, 687 to antisense transcripts distributed between 606 CDS and their UTRs, 67 to tRNAs, and 108 to novel transcripts and CDS. Using the TSS data, the promoter regions and their motifs were analyzed in detail, revealing a conserved -10 (TAnnnT) and a weakly conserved -35 region (nTGACn). The analysis of the 5′ untranslated region (UTRs) of *S. lividans* TK24 revealed 17% leaderless transcripts. Several *cis*-regulatory elements, like riboswitches or attenuator structures could be detected in the 5′-UTRs. The *S. lividans* TK24 transcriptome contains at least 929 operons. The genome harbors 27 secondary metabolite gene clusters of which 26 could be shown to be transcribed under at least one of the applied conditions. Comparison of the reannotated genome with that of the strain *Streptomyces coelicolor* A3(2) revealed a high degree of similarity. This study presents an extensive reannotation of the *S. lividans* TK24 genome based on transcriptome and proteome analyses. The analysis of TSS data revealed insights into the promoter structure, 5′-UTRs, cis-regulatory elements, attenuator structures and novel transcripts, like small RNAs. Finally, the repertoire of secondary metabolite gene clusters was examined. These data provide a basis for future studies regarding gene characterization, transcriptional regulatory networks, and usage as a secondary metabolite producing strain.

## Introduction

The genus *Streptomyces* includes Gram-positive filamentous growing soil bacteria with an extraordinarily high G + C content (Bentley et al., 2002). *Streptomyces* species have a complex life cycle with several biochemical and morphological changes. Spores germinate and form a vegetative mycelium, which in turn produces an aerial mycelium that afterward forms spores again (Leblond et al., 1993).

*Streptomyces* spp. are well known for their potential to produce secondary metabolites such as antibiotics. Important representatives of this genus are *Streptomyces coelicolor* A3(2) and *Streptomyces lividans*. They are the best-characterized *Streptomyces* strains and serve as model organisms (Hopwood, 1999). *S. lividans* TK24 is a plasmid-free, streptomycin-resistant derivative of the strain *S. lividans* 66 (Hopwood et al., 1983) and is often used as a host for cloning or heterologous expression and secretion of proteins or production of enzymes involved in antibiotic production (Hopwood et al., 1995; Bibb, 2005; Hamed et al., 2018).

Due to the wide range of applications, such as secretory production of human proteins (e.g., IL-4R, Zhang et al., 2004), industrial enzymes (Anné et al., 2014; Hamed et al., 2018) and bioethanol production (Lee et al., 2013), *S. lividans* TK24 has become an important strain in the field of biotechnology. Due to their high importance as secondary metabolite producers and the high number of secondary metabolite biosynthetic gene clusters that are apparently inactive or poorly expressed at standard cultivation conditions (Baral et al., 2018), *Streptomycetes* genomes are currently sequenced and explored in a large scale (Harrison and Studholme, 2014). There are 2,340 genomes or draft genomes listed in the NCBI database for the genus *Streptomyces* (NCBI database, 2021).

In contrast to draft genomes, complete *Streptomycetes* genomes are relatively rare, with just 328 listed as complete. The genome sequence of *S. lividans* TK24 was completely determined in 2015 by combining a paired-end whole genome shotgun library and a 7k mate-pair library. Gaps were closed using Sanger sequencing of corresponding PCR products. This version of the genome (GenBank: CP009124.1, submitted: 04-AUG-2014) has a size of about 8.345 Mbp and a G + C content of 72.24%. In total, 7,361 CDS, 18 rRNA genes organized in 6 operons and 64 tRNA genes were predicted (Rückert et al., 2015), with 1,990 CDS annotated as “hypothetical proteins” (Tsolis et al., 2018). Furthermore, 27 putative secondary metabolite gene cluster were predicted by the use of antiSMASH 5.0. By comparison of the *S. lividans* TK24 genome to its close relatives, 507 genes were identified, which have no homologs in *S. coelicolor* A3(2) (Rückert et al., 2015).

The ever-advancing technologies in the post-genomics fields, particularly transcriptomics and proteomics can be used today to generate much more exact descriptions of genes than possible by bioinformatics predictions only.

In this study, the genome of *S. lividans* TK24 was reannotated using a multi-omics approach based on transcriptome and proteome data. For the determination of transcription start sites (TSS) a library enriched for native 5′-ends of transcripts (Pfeifer-Sancar et al., 2013; Irla et al., 2015) and whole transcriptome libraries were sequenced on an Illumina sequencer.

The entire transcriptome information was used to verify and correct rRNA and tRNA predictions, to find novel transcripts and for the correction of translation start sites (TLS). Furthermore, the 5′-UTRs were analyzed regarding ribosome binding sites (RBS) and cis-regulatory elements like riboswitches and leader peptides in attenuated operons. Moreover, promoter motifs were determined using the information of TSS and TLS positions in the genome sequence of *S. lividans* TK24. In addition, secondary metabolite gene clusters were identified, and their transcriptional organization was analyzed. Open reading frames and accurate translation start sites were confirmed by identifying N-terminal peptides in the proteomics dataset. Finally, the genome of *S. lividans* TK24 was compared with that of its close relative *S. coelicolor* A3(2) (Jeong et al., 2016). The updated proteome annotations are accessible through the SToPS database.

## Results and Discussion

### Annotating the Genome in a Multi-Omics Approach by Transcriptome and Proteome Data

**FIGURE 1:**
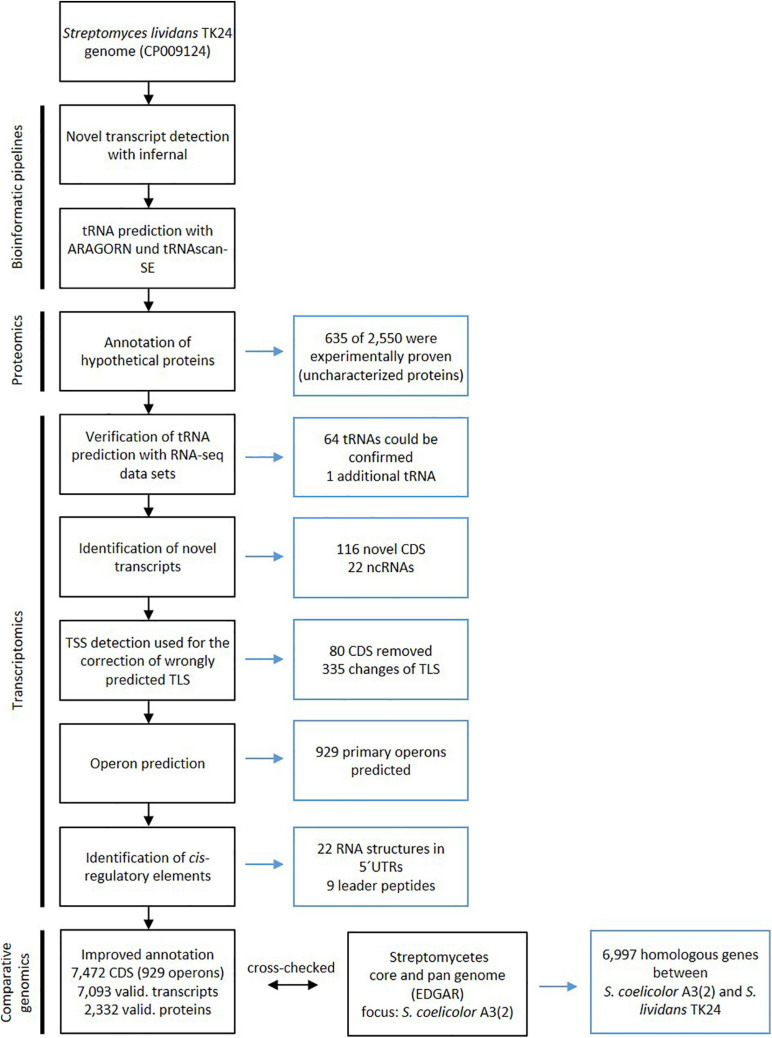
Workflow for improving the *Streptomyces lividans TK24* genome annotation through transcriptomics and proteomics data. On the left side of the flow chart the specific analysis is defined and on the right side the conducted changes are described. *Figure adapted from Wolf et al. (2017), Copyright (2017), with permission from Elsevier*.

To improve the annotation of the *S. lividans* TK24 genome, a combination of transcriptome and proteome data was used. A state-of-the-art bioinformatics pipeline similar to that designed for the reannotation of the *Actinoplanes* sp. SE50/110 genome (Wolf et al., 2017) was applied (Figure 1). Finally, 7,472 CDS were annotated in the *S. lividans* TK24 genome (Table 1), compared to 7,361 predicted CDS in the previous annotation (Rückert et al., 2015).

**TABLE 1:** Features of the reannotated *S. lividans* TK24 genome.

	Previously annotated^1)^	Predicted^2)^	Transcript evidence^3)^	Protein evidence^4)^
No. of CDS	7,361	7,472	7,093	2,332 (307)
Novel CDS*	−	75	64	7
Pseudogenes (predicted)	−	11	5	−
No. of tRNAs	64	84	65	−
No. of rRNAs	18	18	18	−

^1)^ annotation based on Rückert et al. (2015) (accession: CP009124.1, submitted: 04-AUG-2014) ^2)^ automatic prediction by prokka pipeline (Seemann, 2014) ^3)^ Transcript evidence validated with RNA-seq data (this study) with a cut-off of TPM > 1.0 (Busche et al., 2018) ^4)^ Protein evidence was cross-checked with data taken from SToPSdb (http://stopsdb.eu/) (Tsolis et al., 2018); number of proteins identified in this study by their respective N-termini is given in brackets. * Novel CDS that were not annotated in the CP009124.1 genome (submitted: 04-AUG-2014) and were identified by reciprocal best hit BLAST.

Proteomics data were obtained by LC-MS/MS analysis (Tsolis et al., 2019) and used to verify the existence of translated gene products under the tested conditions and to replace annotations of “hypothetical proteins” (Supplementary Table 3). By this, a total number of 2,332 proteins could be detected of which 131 were identified through their respective N-terminus (Table 1). All data were introduced into the SToPSdb database^1^.

**FIGURE 2:**
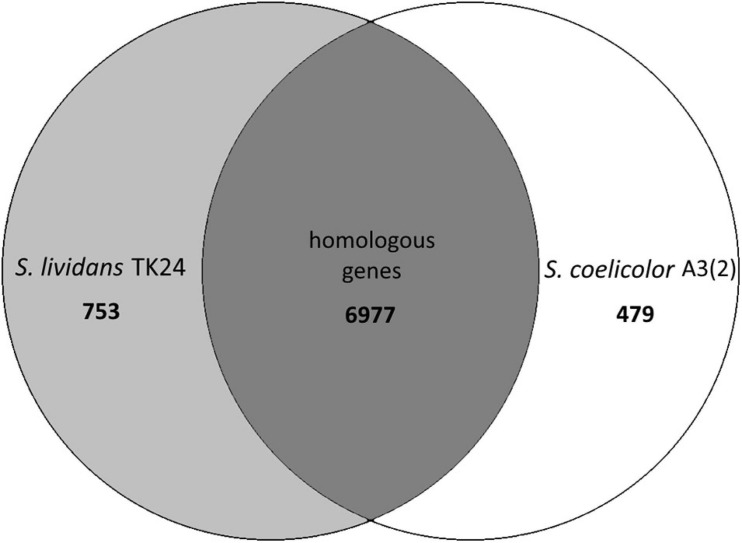
Venn diagram of the number of homologous genes shared between *Streptomyces lividans* TK24 and *S. coelicolor* A3(2), created using EDGAR (Blom et al., 2016), a software platform for comparative genomics.

By comparing the genome of *S. lividans* TK24 (this study) and *S. coelicolor* A3(2) (Jeong et al., 2016) it becomes clear, that the two strains are very similar to each other. The two genomes share ∼92.9% of all their genes (6,977 homologous genes, Figure 2).

### Verification of Automatic tRNA Predictions With RNA-seq Data Sets

The bioinformatic detection of tRNAs in genomes with a high G + C content, like that of *S. lividans* TK24 (72.24%), results in a high false positive rate that is explained by the high G + C content of the tRNAs themselves (Laslett and Canbäck, 2008). The automatic tRNA detection via the prokka pipeline described in Rückert et al. (2015) was verified with the tRNA detection tool tRNAscan-SE 1.21 (Lowe and Eddy, 1997) and the predicted tRNAs were checked for transcription using the whole transcriptome data set.

In this way, all 64 tRNAs predicted by both software tools could be confirmed in addition to one tRNA identified only with ARAGORN.

### Identification of Novel Transcripts in the *S. lividans TK24* Genome

The genome was searched for novel transcripts using the Infernal software (Nawrocki and Eddy, 2013) with Rfam (Nawrocki et al., 2015) as a database. In this context, 23 novel transcripts could be identified containing a tRNA (described above), the tmRNA (RF00023) and 21 small RNAs (sRNA) (Table 2). Among the sRNAs, there is one, which affects *ureB* transcription (RF02514), the 6C RNA (RF01066), one actinobacterial sRNA Ms_IGR-5 (RF02471), one ASdes TB sRNA (RF01781), five ASpks TB sRNAs (RF01782), the RNase P RNA (RF00010), and the bacterial signal recognition particle RNA (RF00169).

**TABLE 2:** Novel transcripts with known functions detected in *Streptomyces lividans* TK24 using the Rfam database.

Name	Locus tag	Rfam ID	RNA-seq validation^1^	Sequence position
ASdes TB sRNA	SLIV_04927	RF01781	no	1,074,703
ASpks TB sRNA	SLIV_06772	RF01782	no	1,528,116
ASpks TB sRNA	SLIV_06774	RF01782	no	1,533,336
ASpks TB sRNA	SLIV_06777	RF01782	no	1,538,983
ASpks TB sRNA	SLIV_06779	RF01782	no	1,544,263
ASpks TB sRNA	SLIV_06782	RF01782	no	1,549,767
Streptomyces RNA 6106	SLIV_07607	RF02827	yes	1,749,289
Streptomyces RNA 5676	SLIV_10247	RF02832	yes	2,368,920
Streptomyces sRNA scr5239	SLIV_12172	RF02605	yes	2,796,320
Streptomyces sRNA scr5239	SLIV_14952	RF02673	no	3,380,997
Actinobacterial sRNA Ms_IGR-5	SLIV_16488	RF02471	yes	3,696,473
Streptomyces RNA 4115	SLIV_17827	Scr4115	yes	3,971,962
Bacterial small signal recognition particle RNA	SLIV_18113	RF00169	yes	4,030,118
Streptomyces RNA 3920	SLIV_18667	RF02828	yes	4,145,142
6C RNA	SLIV_20498	RF01066	yes	4,531,561
Streptomyces RNA 3202	SLIV_21682	RF02833	no	4,831,696
transfer-messenger RNA	SLIV_22873	RF00023	yes	5,115,155
Streptomyces RNA 2736	SLIV_23991	RF02831	no	5,351,661
sodF sRNA	SLIV_24507	RF02790	yes	5,474,012
Bacterial RNase P class A	SLIV_26257	RF00010	yes	5,877,232
Streptomyces RNA 1601	SLIV_29722	RF02830	yes	6,628,022
5′ ureB small RNA	SLIV_31627	RF02514	no	7,030,980

*^1^ “yes”: transcribed under the analyzed conditions; “no”: not transcribed under tested conditions*

The transcription of 12 of these novel transcripts could be verified by analyzing the RNA-seq data. This includes the bacterial RNase P class A (*SLIV_26257*), the bacterial signal recognition particle RNA (*SLIV_18113*), and the tmRNA (*SLIV_22873*), which are conserved in all bacteria, as well as the 6C RNA (*SLIV_20498*), which is conserved in all actinobacteria.

### Improving the Annotation of Coding Regions by Correcting Wrongly Predicted Translation Start Sites

Due to the high G + C content of the *S. lividans* TK24 genome and the resulting fewer AT-rich stop codons, N-terminally extended ORFs are erroneously predicted (Hyatt et al., 2010). For this reason, translational start sites were manually corrected using the TSS predictions resulting from the RNA-seq data (Supplementary Table 2). In total, 335 start codons were changed, of which 48% were wrongly predicted ATG, 46% GTG and 6% TTG start codons. After TLS correction, the total amount of ATG start codons was increased to 55.4%, whereas the number of GTG and TTG start codons was decreased to 40.8% and 3.9% respectively.

Furthermore, 80 CDS were removed due to disagreements in the RNA-seq data (e.g., transcription on the opposite strand) and 116 novel CDS were annotated (Supplementary Table 1). About 26.7% of the novel annotated CDS are transcribed leaderless, and 30.2% have a 5′UTR. For the remaining 46.5% of the novel CDS, no TSS could be assigned or the genes are located in an operon. For 78 of the 116 novel CDS (67.2%) a homologous protein-coding gene in the genome of *S. coelicolor* A3(2) could be found by BLASTX search (Altschul et al., 1997).

### Identification of Transcription Start Sites Through RNA-seq of 5′-Ends of Native Transcripts

To ensure a high annotation quality of the *S. lividans* TK24 genome, an RNA-seq data set enriched for native 5′-transcript ends and whole transcriptome datasets were used for the refinement. The sequencing of the 5′-transcript end library revealed 2.5 million reads mapping to the *S. lividans* TK24 genome. For the whole transcriptome sequencing around 191 million reads were mapped on the genome. For this, transcriptomes were sequenced from two cultivations in two different media and at three different time points as described previously (Tsolis et al., 2019). This data set can be used to identify transcribed (novel) genes, operon structures and small or antisense RNAs.

**FIGURE 3:**
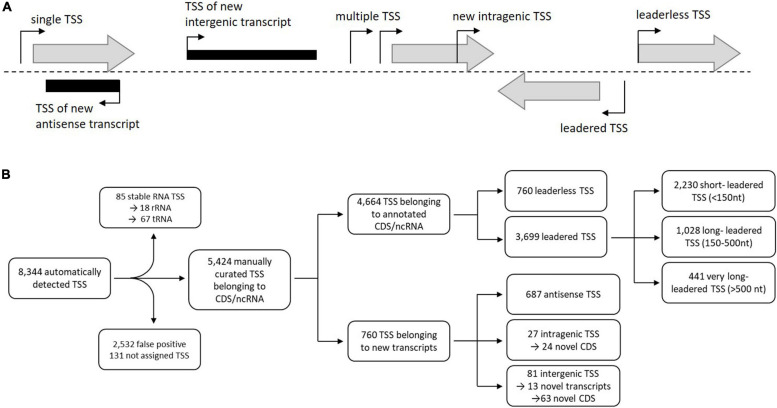
**(A)** Classification of transcription start sites (TSS) based on genomic context (expanded from Pfeifer-Sancar et al., 2013). The first TSS classification level is divided into two categories: TSSs that belong to annotated genes (gray shaded arrows) and TSSs that belong to new transcripts (black rectangles). TSSs belonging to annotated genes were classified into single TSSs or multiple TSSs. TSSs belonging to new transcripts were arranged into antisense, intragenic, or intergenic TSSs. **(B)** Identification, filtering, and classification of TSSs (expanded from Pfeifer-Sancar et al., 2013). From the 8,344 automatically detected TSSs, those TSSs were removed that belong to rRNAs or tRNAs (85) as well as those determined to be false-positive by manual inspection (2,532) for further analyses resulting in 5,580 putative TSS assigned to different transcript types.

Transcription start sites (TSS) were identified using the 5′-end RNA-seq data set (Figure 3). The analysis and visualization was carried out with the software ReadXplorer 2 (Hilker et al., 2016) as described by Wolf et al. (2017). 8,344 TSS were detected by automated prediction, of which 343 TSS are located in rRNA and tRNA genes and 2,532 false positive TSS were excluded from further analysis. Finally 5,580 manually curated TSS of which 5,424 TSS belonging to annotated genes and 760 belonging to new transcripts (Figure 3B, Supplementary Table 4). 687 TSS of these were classified as putative antisense TSS due to their orientation to 606 CDS and their UTRs. 108 TSS could be assigned to novel CDS or novel transcripts (Figure 3B, Supplementary Table 1). Furthermore, the RNA-seq datasets were used to verify and to correct the translation start sites for the improvement of the annotation, to verify the tRNA prediction, for the identification of novel transcripts and small RNAs, as well as for the identification and analysis of cis-regulatory elements like riboswitches (Table 3).

**TABLE 3:** *Cis*-regulatory elements detected in the *Streptomyces lividans* TK24 genome by searching the Rfam database and validated by RNA-seq data.

*Cis*-regulatory element	Rfam ID	Affected transcripts	Related function	Sequence position
Actino-pnp RNA	RF01688	(Actino-pnp)-*pnp*	RNA degradation	2,286,204
Che1 RNA	RF02935	(che1)-*SLIV_11405*	Cholesterol metabolism	2,630,791
Cobalamin riboswitch	RF00174	(Cobalamin)-*cbiM*-*SLIV_08815-SLIV_08820-SLIV_08825*	Cobalt transport	2,013,427*
Cobalamin riboswitch	RF00174	(Cobalamin)-*cobD*-*cobQ-cobN-SLIV_28465*	Cobalamin biosynthesis	6,366,946
FMN riboswitch	RF00050	(RFN element)-	Riboflavin biosynthesis	6,799,666
		*SLIV_30550-*		
		*SLIV_30555-*		
		*SLIV_30560*		
Glycine riboswitch	RF00504	(Glycine)-*gcvT-gcvH*	Glycine cleavage	2,561,721
Glycine riboswitch	RF00504	(Glycine)-*gcvP*	Glycine cleavage	6,881,113
msiK RNA	RF01747	(*msiK*)-*sugC*	Uptake of several kinds of (complex) sugars	3,846,034
NrdJ RNA	RF03034	(nrdJ)-*nrdJ*	Deoxyribonucleotide biosynthesis	2,198,217
rai-hairpin	RF03059	(rai)-SLIV22665	Transcriptional modulation	5,058,391
S-adenosyl methionine (SAM) IV riboswitch	RF00634	(SAM-IV)-*SLIV_26975*	Cysteine desulfurase (Thiamine metabolism)	6,033,447
SAM riboswitch (S box leader)	RF00162	(SAM)-*SLIV_16930-SLIV_16925*	Threonine biosynthesis	3,785,632
Streptomyces-metH RNA	RF03063	(Streptomyces-metH RNA)-*metH*	Methionine biosynthesis	6,566,055
Streptomyces-metK RNA	RF03076	(Streptomyces-metH RNA)-*metK*	SAM biosynthesis	6,761,134
TPP riboswitch (THI element)	RF00059	(TPP)-*SLIV_27170-SLIV_27165-thiG*	Thiamine biosynthesis	6,077,630
TPP riboswitch (THI element)	RF00059	(TPP)-*SLIV_10395-SLIV_10390-fbpC-SLIV_10380*	Thiamine transport	2,401,194
ydaO/yuaA leader	RF00379	(*ydaO-yuaA*)-*SLIV_04650*	Secreted hydrolase - Peptidase (Sec)	1,014,942
ydaO/yuaA leader	RF00379	(*ydaO-yuaA*)-*SLIV_09410*	Secreted hydrolase - Peptidase (Sec)	2,153,832
ydaO/yuaA leader	RF00379	(*ydaO-yuaA*)-*SLIV_14380*	Secreted hydrolase - Enodpeptidase, NLPC/P60 domain (IPR000064) (Sec)	3,273,523
ydaO/yuaA leader	RF00379	*(ydaO-yuaA)-SLIV_15360*	Transglycosylase	3,469,099
ydaO/yuaA leader	RF00379	(*ydaO-yuaA*)-*SLIV_17860*	Secreted hydrolase - Cell wall peptidase (Sec)	3,980,097
ydaO/yuaA leader	RF00379	(*ydaO-yuaA*)-*SLIV_22200*	Secreted hydrolase - Protein with Lysozyme-like domain (IPR023346) and LysM domain (IPR018392) (Sec)	4,948,999

* not transcribed under tested conditions.

In *S. coelicolor* A3(2) the identification of transcription start sites revealed a total of 3,570 TSSs, which were further categorized into primary (2,771), secondary (333), antisense (256), intragenic (79) and intergenic (131) TSSs (Jeong et al., 2016).

### Genomic Features Deduced From the Location of Transcription Start Sites of the *S. lividans* TK24 Genome Sequence

#### Analysis of the *S. lividans* TK24 Transcripts With and Without 5′-UTR

**FIGURE 4:**
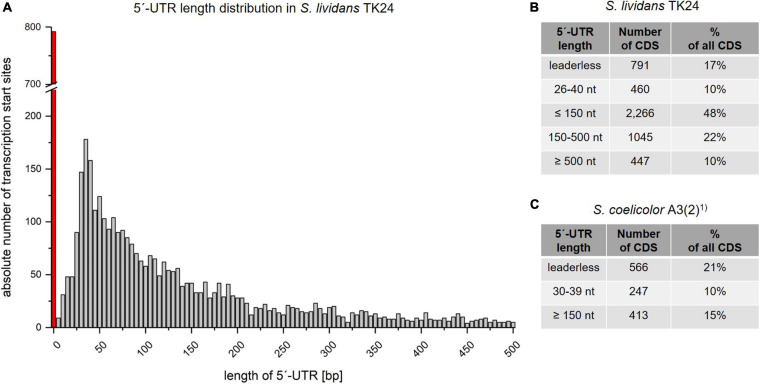
Length distribution of the 5′-untranslated regions (5′-UTRs). **(A)** 5′-UTR length distribution based on 4,717 identified TSSs and up to a length of 500 nucleotides. X axis: Absolute number of leader sequences detected for the given length interval. Y axis: The length of leader sequences plotted in 5 nucleotide intervals. The red bar represents leaderless transcripts with a leader sequence length of 0 to 3 nucleotides. **(B)** Different categories of 5′-UTR lengths in *Streptomyces lividans* TK24. **(C)** Different categories of 5′-UTR lengths in *S. coelicolor* A3(2), based on the data of Jeong et al. (2016)^1)^.

Bacterial mRNAs most often have a 5′untranslated region (5′-UTR), which differ in length between different transcripts. These UTRs are often called leader transcripts and play an important role in the regulation of transcription and translation. Such a leader also contains the ribosome binding site. Transcripts with no 5′-UTR are called *leaderless*. The analysis of all transcripts of *S. lividans* TK24 revealed the size distribution of their 5′-UTRs (Figure 4). The UTR length varies between 0 and 3,068 nucleotides, with 90% of all analyzed UTRs having a length of less than 500 nt. More than 75% of the leader transcripts are 5-200 nucleotides long.

For most bacteria analyzed by bioinformatics predictions, the most common 5′-UTR length was less than 30 (Sorek and Cossart, 2010) to 40 nucleotides (Passalacqua et al., 2009). However, this estimation may change due to recent developments in NGS technologies (McClure et al., 2013). Leaderless transcripts in the *S. lividans* TK24 genome were determined to be 791 (17% of all primary TSS) (Figures 3B, 4B), which is in common with the proportion of leaderless transcripts identified in *S. coelicolor* A3(2) (566; 21.0%) by Jeong et al. (2016) (Figure 4C). These numbers include not only monocistronic genes but also the first genes of operons. ATG and GTG translation start codons were found a frequency of 67.8% and 32.2%, respectively, in all leaderless transcripts. The most frequent 5′-UTR length range is 26–40 nt (460 10%) in *S. lividans* TK24, which matches the findings for *S. coelicolor* A3(2) (30-39 nt; 247; 10%) (Figures 4B, 4C).

#### Global Identification of −10 and −35 Promoter Consensus Motifs in the *S. lividans* TK24 Genome

Based on the 5**′**-end transcriptome library data and the resulting exact position of the TSS, we were able to search for promoter motifs, such as the −10 region (Pribnow box) and the −35 region of promoters addressed by the house-keeping sigma factor.

**FIGURE 5:**
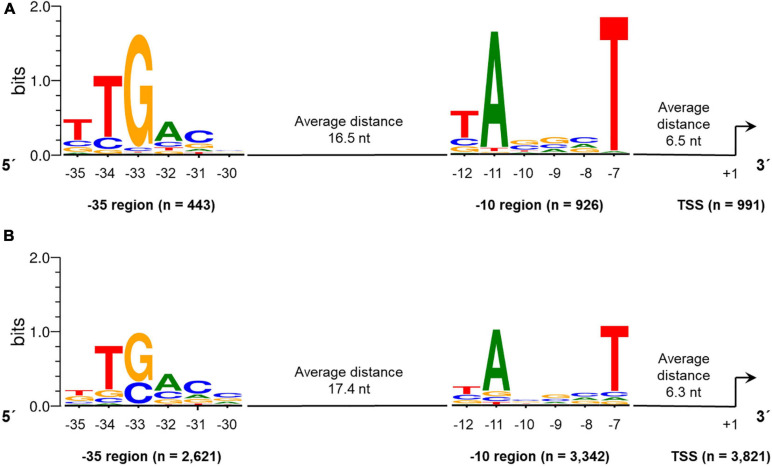
Conserved –10 and –35 regions identified in promoter regions of leaderless **(A)** and leadered **(B)** transcribed genes in the *Streptomyces lividans* TK24 genome. The motifs were identified using Improbizer (Ao et al., 2004), the logos were created using WebLogo (Crooks et al., 2004).

Therefore, 70 bases upstream of the identified primary TSS were searched with the web tool Improbizer (Ao et al., 2004). For the −10 region a conserved hexamer motif represented by TAnnnT was found in 88.7% of all sequences examined (4,268 of 4,812 sequences in total) (Figure 5 and Supplementary Table 5). For leaderless transcribed genes, the conserved -10 sequence was found in 93.4% of all sequences. The T on the first position of the identified hexamer was found in 63.8% of the leaderless and in 42.9% of the analyzed leadered sequences. For the A on second position within the −10 motif, a frequency of 94.5% in leaderless and 70.6% of leadered transcribed genes could be determined. In the last position of the -10 hexamer, a T could be identified with an abundance of 97.4% and 73.6% in the considered sequences of leaderless and leadered transcribed genes in *S. lividans* TK24.

The average distance between the 10 hexamer and the TSS was 6.3 nucleotides (Figure 5). The distance ranges between 4 and 9 nucleotides whereas 88.7% of all spacers are between 5 and 7 nucleotides in length. For 97.1% of the leaderless and 72.3% of the analyzed leadered TSS, the first base is a purine (A or G).

Next, the aligned sequences were used for the analysis of the −35 region using Improbizer (Figure 5). The distance between −10 and −35 was restricted to a length of 15 to 19 nucleotides, resulting in a total number of 443 (leaderless) and 2,621 (leadered) examined sequences. The consensus hexamer motif was TTGACn for the leaderless and nTGACn for the leadered transcribed genes respectively, since the T on the first position was not identified frequently for leaderless transcripts (Supplementary Table 5). These are largely consistent with the *E. coli* −35 consensus motif TTGACA. This motif could be identified in 3,064 of all 4,812 analyzed sequences (63.7%). The average distance between the −10 and the −35 regions was found to be 16.5 nt (leaderless) and 17.4 nt (leadered), which is similar to the spacer length of 17 nt described as optimal in *E. coli* (Singh et al., 2011).

In *S. coelicolor* A3(2) the -10 motif was found to be TAnnnT (in 80.4% of the analyzed TSS upstream regions, which is identical to the identified consensus sequence in this study. In addition, the −35 region of *S. coelicolor* A3(2) (nTGACC; upstream of 58.6% of the TSSs) is very similar to that found in *S. lividans* TK24 (TTGACn; Figure 5). The slight difference in the sixth position may be due to the different number of analyzed TSS or different weighting criteria.

#### Determination of Ribosome Binding Sites in the *S. lividans* TK24 Genome Sequence

**FIGURE 6:**
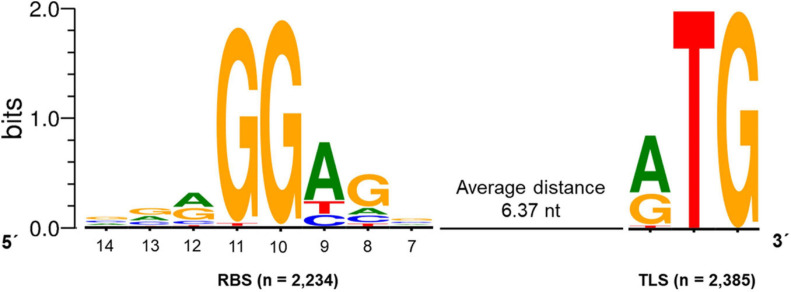
Conserved ribosomal binding site (RBS) motif of *Streptomyces lividans* TK24. The consensus sequence ^*A*^/_*G*_GGAGn was found in 2,234 sequences upstream of the translation start (TLS) codon identified in 2,385 CDSs with an experimentally determined primary TSSs and a 5′-UTR of 10-150 nt. The average distance between the RBS and the TLS was 6.37 nt. The conserved RBS motif was identified using Improbizer (Ao et al., 2004), the logo was created using WebLogo (Crooks et al., 2004).

For the identification of potential ribosome binding sites (RBS; Shine-Dalgarno sequence) in *S. lividans* TK24, all CDS with a primary TSS and a 5′-UTR size of 10 to 150 nucleotides were analyzed. For those CDS (2,385), the sequence 20 nucleotides upstream of the start codon was searched for a conserved RBS motif using the web tool Improbizer (Ao et al., 2004). In 93.7% of all analyzed sequences (2,234 out of 2,385) a conserved RBS motif could be found. The detected consensus motif is ^*A*^/_*G*_GGAGn (Figure 6) with an average distance to the translation start codon of 6.37 nt. The spacer length ranged between 4 and 9 nt.

The first position of the identified hexamer was determined to be an A or a G (45.1% and 36.5%, respectively). G was commonly found in position 2 and 3 with a frequency of 97.4% and 98.6%, followed by an A in position 4 identified in 70% of the sequences and a G in position 5 found in 64.1% of all analyzed sequences. The sixth position base was not conserved.

The identified motif fits to sequence AGGAGG described in previous studies (Burger et al., 1998) and was found to increase gene expression compared to modified ribosomal binding sites in *S. coelicolor* A3(2) (Luo et al., 2017). The determined RBS consensus sequence ^*A*^/_*G*_GGAGn partly matches the 3′-end of the 16S rRNA 3′UUU**CCUC**CA5′ found in the *S. lividans* TK24 genome.

Furthermore, the motif is very similar to the RBS/Shine-Dalgarno sequence AGGAGG described for *E. coli* (Shine and Dalgarno, 1974; Omotajo et al., 2015). The spacer length between the RBS and the translation initiation codon is in the range of 5–10 nt, which has been described to be optimal for efficient translation initiation in the Bacteria (Omotajo et al., 2015). The *E. coli* RBS was found 8-13 nucleotides upstream of the TLS with an average distance of 6.9 (Stormo et al., 1982). The spacer length for the strongest translation efficiency was determined to be 8 nt in *E. coli* (Ringquist et al., 1992). Here, the spacer length for *S. lividans* TK24 was found to be 6.37 nt, which is shorter compared to *E. coli*. This difference could be due to the slightly shorter 16S rRNA in *S. lividans* TK24 (1,514 nt) compared to *E. coli* (1,542 nt) (Brimacombe, 1978).

For *S. coelicolor* A3(2) a conserved polypurine (G > A) region 8-12 bp upstream of the TLS was described, which was found in 2,139 5′-UTR sequences. The spacer length of this identified region ranges between 5–8 nt. (Jeong et al., 2016).

#### Analysis of 5′-UTRs for *Cis*-Regulatory Elements

The bioinformatic analysis of the 5′-UTR (leader sequences) of *S. lividans* TK24 revealed a number of different predicted *cis*-regulatory elements (Table 3). Using RNA-seq data and the results of the TSS detection analysis, different riboswitches and other *cis*-regulatory elements were validated and characterized with respect to their transcriptional boundaries.

The validation of those elements was based on characteristic transcription profiles. In many cases of premature transcriptional termination through riboswitches, an increased number of mapped reads in the 5′-UTR compared to the downstream CDS could be observed under suitable conditions (Rosinski-Chupin et al., 2014). The analysis of the 5′-UTRs using the Rfam database revealed 22 different *cis*-regulatory elements with an *E*-value of less than 0.01, many of which belong to riboswitches (Table 3). Using the RNA-seq data it could be shown that 21 of the 22 cis-regulatory elements were highly transcribed within in the 5′-UTR of the downstream gene, validated by the identified upstream TSS and the corresponding TLS of the downstream transcribed gene. This strongly suggests that they would regulate these downstream genes.

Riboswitches play an important role in premature transcriptional termination. Specific environmental signals are sensed through small metabolites, which interact with the leader RNA and form alternative terminator or anti-terminator structures (Naville and Gautheret, 2010a; Millman et al., 2017). Alternatively, riboswitches affect the translational initiation through a blockade of the ribosome binding site by forming alternative stem loop structures in this area (Waters and Storz, 2009; Abduljalil, 2018).

For all cis-regulatory elements in Table 3, a biological function could be assigned to the likely affected downstream coding region. These include two cobalamin (vitamin B12) binding riboswitches (RF00174) identified upstream of the *cobD*, *cobQ* and *cobN* genes, which are involved in cobalamin biosynthesis (Nahvi et al., 2004; Peselis and Serganov, 2012) and upstream of genes related to cobalt transport (Fowler et al., 2010).

Furthermore, glycine riboswitches (RF00504) were found upstream of the genes *gcvT*, *gcvH*, and *gcvP* coding for a glycine cleavage system (Mandal et al., 2004). A thiamine (vitamin B1) riboswitch (RF00059) was identified in the 5′-UTR of genes involved in thiamine transport. These riboswitches bind the activated form thiamine pyrophosphate (TPP), an essential coenzyme in prokaryotes (Hohmann and Meacock, 1998; Serganov et al., 2006).

In addition, two different variants of S-adenosylmethionine (SAM)-dependent riboswitches were identified. The first one is a SAM-IV riboswitch (RF00634) upstream of gene *SLIV_26975* coding for a cysteine desulfurase (Grundy and Henkin, 1998). The second one is a SAM (S box leader) riboswitch (RF00162), which was found upstream of genes involved in threonine biosynthesis (Serganov and Patel, 2009).

Additionally, a *msiK* motif (RF01747) regulating genes encoding a sugar uptake system, which has also been described for *S. coelicolor* A3(2) (Bertram et al., 2004), as well as an actino-*pnp* RNA (RF01688) in the 5′-UTR of the polynucleotide phosphorylase gene *pnp* itself (Jarrige et al., 2001) were predicted and validated in the genome of *S. lividans* TK24.

Finally, six leaders with a *ydaO/yuaA* riboswitch were predicted in the genome of *S. lividans* TK24. The *ydaO/yuaA* riboswitch is also called cyclic di-AMP riboswitch, as it senses the cyclic di-AMP level inside the cell and is therefore related to stress, e.g., DNA damage or cell wall stress (Barrick et al., 2004; Nelson et al., 2013). The genes typically regulated by *ydaO/yuaA* riboswitches in Actinobacteria are connected to cell wall metabolism (Nelson et al., 2013), like the cell wall peptidase SLIV_17860. Five of the leaders with a *ydaO/yuaA* riboswitch were shown to be transcribed under the cultivation conditions which were applied in this study. Each of the genes they regulate encodes for secreted proteins, like hydrolases, a transglycosylase and an endopeptidase.

In *S. coelicolor* the *ydaO/yuaA* riboswitch was also found upstream of genes encoding cell wall hydrolases, which affect germination, sporulation, and vegetative growth (Haiser et al., 2009). In other Gram-positive species like *Bacillus subtilis* it was shown, that increasing cyclic di-AMP level leads to transcriptional termination (Nelson et al., 2013), but in *S. coelicolor* it seems that the riboswitch acts on translation initiation by building a stem-loop structure and blocking the RBS of the downstream hydrolase genes (Haiser et al., 2009; Block et al., 2010). Based on this, the same regulation can be assumed for *S. lividans* TK24.

The 5′UTR of the genes, which are involved in the biosynthesis and metabolism of amino acids were analyzed for short leader peptides (Table 4). These leader peptides are often enriched in codons for the corresponding amino acids biosynthesized by the enzymes of the downstream operon to form small peptide ORFs and comprise regions with the potential of forming competing hairpins. Attenuators are a regulatory mechanism which is based on the coupling between transcription and translation in which the latter controls the former (Naville and Gautheret, 2010b). Ribosomal stalling or fast sliding over the leader peptide ORF biases the formation either of transcription termination or anti-termination hairpins, that this allows or prevents the transcription of biosynthetic genes that lie downstream (Vitreschak et al., 2004). This way, the cell finely regulates expensive anabolic resources. In the genome of *S. lividans* TK24, 8 leader peptides were identified with two being leucine-dependent (*leuL*), two tryptophan-dependent (*trpL*) and one each depending on alanine (*alaL*), isoleucine (*ilvL*), methionine (*metL*) and threonine (*thrL*) (Table 4). The two leucine-dependent leader peptides are located upstream of the *leuS* (leucine-tRNA ligase) and the *leuA* (2-isopropylmalate synthase) genes. LeuS loads leucine onto the corresponding tRNA. The 12 amino acids long LeuL2 leader peptide contains three leucines (MRAVR**LLL**SEPR^∗^). It is transcribed leaderless and is followed by a hairpin structure and a stretch of uridines, which seem to promote Rho-independent transcriptional termination. The 15-residue-long LeuL1 leader peptide contains five leucines (MRFG**LLLL**SCRGEG**L**^∗^). It is transcribed leaderless but lacks a typical Rho-independent transcriptional terminator. This was also described for leader peptides upstream of the *leuA* gene in other *Actinobacteria* (Seliverstov et al., 2005; Neshat et al., 2014; Wolf et al., 2017).

**TABLE 4:** Attenuator structures identified through RNA-seq with the affected transcripts and the translated leader peptide with the amino acids initiating the attenuation marked in bold.

Attenuator	Affected transcripts	Description	Leader peptide	Sequence position
*alaL* (*SLIV_01313*)	*alaS* (*SLIV_01315*)	Alanyl-tRNA synthetase	MNVIGRNIF**A**T**A**R**A**TSSPVAAA*	270,351
*ilvL* (*SLIV_10947*)	*ilvBHC* operon	Biosynthesis of Val, Leu, Ile	MRTR**ILVL**GKRVG*	2,517,781
*thrL* (*SLIV_19412*)	*thrS* (*SLIV_19410*)	Threonine-tRNA ligase	MKRVRPFLE**TT**PGFVPAR*	4,312,812
*trpL2* (*SLIV_21112*)	*trpS* (*SLIV_21110*)	Tryptophan-tRNA ligase	MMTRTCTQL**W**RAA*	4,665,006
*leuL2* (*SLIV_24828*)	*leuS* (*SLIV_24830*)	Leucine-tRNA ligase	MRAVR**LLL**SEPR*	5,555,105
*leuL1 (SLIV_25043)*	*leuA* (*SLIV_25045*)	2-isopropylmalate synthase	MRFG**LLLL**SCRGEGL*	5,606,355
*trpL1 (SLIV_27122)*	*trpE* (*SLIV_27120*)	Anthranilate synthase	MFAHSTRN**WWW**TAHPAAH*	6,065,805
*metL* (*SLIV_29928*)	*metNIQ* operon	Methionine import system	MSTTSDRTPATEATTTPGARC**M**CRR**M**CAF*	6,668,039

The * denotes a stop codon.

The two tryptophan-dependent leader peptides *trpL1* and *trpL2* are located upstream of the genes *trpE* (encoding for anthranilate synthase) and *trpS* (encoding for tryptophan-tRNA ligase). TrpE is involved in an early step of tryptophan biosynthesis. Its leader peptide TrpL1 consists of 18 amino acids including 3 tryptophans (MFAHSTRN**WWW**TAHPAAH^∗^), is transcribed leaderless and its transcriptional termination seems to be Rho-independent due to the presence of two hairpin structures and a uridine enriched stretch between *trpL1* and the monocistronic *trpE* gene. However, TrpL2 consists of 13 amino acids including only one tryptophan (MMTRTCTQL**W**RAA^∗^). The transcription start is also leaderless, and the termination is induced by two hairpin structures, but no uridine-enriched sequence was found downstream of *trpL2*.

Trp-dependent leader peptides are found in several bacteria. They are often located upstream of the *trp* biosynthesis operon (Kolter and Yanofsky, 1982). However, in *S. lividans* TK24 these two attenuator structures are located upstream of the two monocistronically transcribed genes *trpE* and *trpS*.

The AlaL leader peptide is located upstream of the *alaS* gene encoding an alanyl-tRNA synthetase. This enzyme is responsible for the attachment of the appropriate amino acid onto its tRNA. The leader peptide consists of 22 amino acids including 6 alanines (MNVIGRNIF**A**T**A**R**A**TSSPV**AAA**^∗^). This type of leader peptide has not been described in bacteria before.

The attenuator sequence encoding the leader peptide IlvL is located upstream of the operon *ilvBHC*, which encodes key enzymes in the biosynthesis of the branched-chain amino acids valine, isoleucine, and leucine. The leader peptide with a length of 13 amino acids (MRTR**ILVL**GKRVG^∗^) is transcribed leaderless and its transcriptional termination seems to be Rho-independent due to several hairpin structures and a uridine-rich sequence region downstream of the *ilvL* gene. It contains one codon for valine and isoleucine as well as two for leucine. This shows that these amino acids are involved in the regulation of their own biosynthesis as shown in several Actinobacteria (Seliverstov et al., 2005; Neshat et al., 2014; Wolf et al., 2017) and in *S. coelicolor* A3(2) (Craster et al., 1999).

A further attenuator structure was identified upstream of the gene *metN*, which is part of the methionine-dependent import system MetNIQ. The leader peptide has a length of 29 amino acids (MSTTSDRTPATEATTTPGARC**M**CRR**M**CAF^∗^) and contains several methionines and threonines. The transcription start of *metL* is leaderless, and its transcriptional termination is unclear. Whereas there are several hairpin structures downstream of *metL*, a typical structure of Rho-independent termination is missing.

Finally, a threonine-dependent leader peptide sequence could be identified upstream of the *thrS* gene encoding a threonine-tRNA ligase. The leader peptide consists of 18 amino acids (MKRVRPFLE**TT**PGFVPAR^∗^) containing two threonines. The transcription of *thrL* starts leaderless, whereas its transcriptional termination is not clear, since one hairpin but no uridine rich sequence, necessary for a clear Rho-independent termination (Gusarov and Nudler, 1999), were found downstream of *thrL*.

### Identification of Operon Structures by Combining the 5′-End and the Whole Transcriptome Data Sets

Two or more genes that are transcribed from a single promoter form an operon. Operon detection was performed using the software ReadXplorer2 (Hilker et al., 2016). The data of all six RNA-seq experiments were combined to increase the number of reads in regions with low coverage. The identified primary operons were checked for experimental validation using the TSS. If an operon has an assigned TSS, it is experimentally validated, if not, it was specified as predicted operon. The class of sub-operons consists of operons which show a TSS for a posterior gene in a primary operon. All other genes, which could not be connected to an operon, were assigned to be monocistronically transcribed.

**FIGURE 7:**
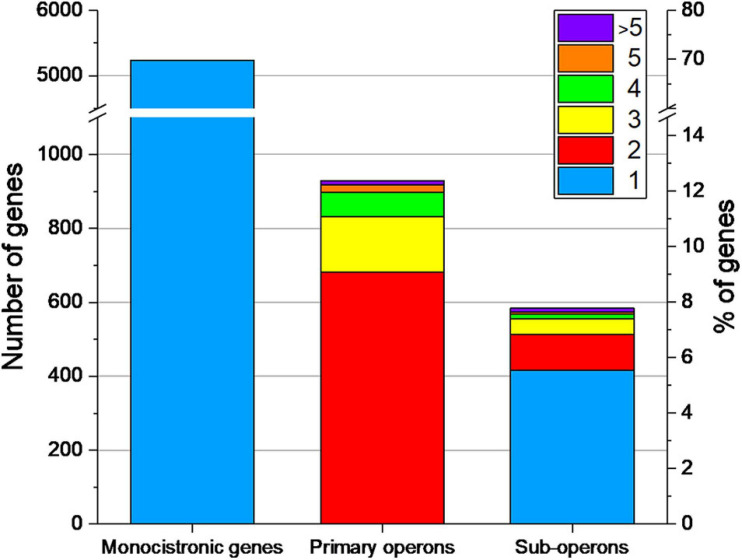
Number of monocistronic genes, and genes located in primary operons or sub-operons of *Streptomyces lividans* TK24. The number of genes included in primary and sub-operons is color-coded.

Under the studied conditions 929 primary operons containing 2,274 genes (30.3% of the genome) could be determined by combining the 5′-end and the whole transcriptome data sets (Figure 7).

Of the primary annotated operons 444 (48%) could be experimentally validated, as a TSS could be assigned to their first gene. By analyzing the internal TSS, 584 sub-operons could be determined inside the 929 primary operons. The majority of the sub-operons consists of a single gene.

The largest primary operon contains 14 genes, which encodes the sub-units of NADH-quinone oxidoreductase, an enzyme responsible for electron shuttling in the respiratory chain (Sousa et al., 2012).

The number of monocistronically transcribed genes was determined to be 5,234 (70.0% of all CDS), of which about a half (2,639 genes) were associated with a TSS in this study.

### The Transcriptional Organization of Secondary Metabolite Gene Clusters

Secondary metabolite gene clusters are very common in bacteria, especially in the genus *Streptomyces*. Many of these metabolites have interesting biological properties and show structural and functional diversity (Osbourn, 2010). Secondary metabolites have been exploited for a long time as antibiotics or anticancer agents in medicine and agriculture (Seca and Pinto, 2018). Due to progress in next-generation sequencing techniques, mining bacterial genomes for the identification of novel gene clusters is a promising approach to find novel compounds of biological and medical importance (Adamek et al., 2017).

For identification of novel biosynthetic gene clusters in the *S. lividans* TK24 genome, we applied the antiSMASH 5.0 tool (Blin et al., 2019). The analysis revealed 27 potential gene clusters (Table 5), all of which were also identified in the genome of the closely related strain *S. coelicolor* A3(2) (Bentley et al., 2002). Several of these gene clusters have been described in closely related *Streptomyces* sp., like the actinorhodin gene cluster (Okamoto et al., 2009) or the prodiginine gene cluster in *S. coelicolor* A3(2) (Williamson et al., 2006) and the coelimycin (*cpk*) gene cluster in *S. coelicolor* M145 (Gomez-Escribano et al., 2012). 26 of the 27 identified gene clusters show expression under one of the tested growth conditions in this study. A polyketide synthase (PKS) type I gene cluster for the potential production of coelimycin was identified but shows only very low expression levels under the different conditions tested. Coelimycin occurs as a yellow pigment but the function of this polyketide is still unclear. Its complex regulation was recently described in *S. coelicolor* A3(2) (Bednarz et al., 2019). The strongest expression was shown for the siderophore gene cluster, responsible for desferroxamine B production, and the ectoine biosynthetic gene cluster. Desferroxamine is a strong siderophore, which is used in the treatment of iron poisoning. It is mainly produced by *Streptomyces pilosus* (Chiani et al., 2010). Ectoine is a osmoprotectant which is produced by several bacteria to survive extreme salt concentration. It was found in both Gram-negative and Gram-positive bacteria (Peters et al., 1990). In *S. coelicolor* A3(2) production of actoine was detected under salt-induced osmotic stress. Ectoine production could be even increased by the addition of ectoine into the medium (Bursy et al., 2008).

**TABLE 5:** Biosynthetic gene clusters predicted in the *Streptomyces lividans* TK24 genome through antiSMASH 5.0 (Blin et al., 2019) and the transcription of the clusters analyzed by RNA-seq.

Cluster number	Gene cluster type	Putative product	Sequence position		Transcription strength^1)^	Described in *S. coelicolor* A3(2)^2)^	References
			**From**	**To**			
1	Terpene	Albaflavenone	17,147	36,814	±	no	Zhao et al., 2008
2	NRPS	Coelibactin	145,846	218,924	+ +	yes	Komatsu et al., 2008; Jeong et al., 2016
3	Indole	Indole	408,558 -	429,567	+	no	Zhao et al., 2012; Jeong et al., 2016
4	Other	germicidin	651,233	724,117	n.d.	no	
5	Terpene	Hopanoids	1,013,842	1,039,649	+ +	yes	Jeong et al., 2016
6	Lantipeptide	Lanthionine-containing peptide SapB	1,112,999	1,135,713	+	yes	
7	NRPS	Non-ribosomally synthesized dipeptide	1,373,403	1,427,601	+	yes	this study
8	PKS (Type I)	Butyrolactone (Coelimycin P1)	1,504,474	1,574,690	−	yes	this study
9	Siderophore	Siderophore	1,601,584	1,613,301	±	no	Poralla et al., 2000; Seipke and Loria, 2009; Liu et al., 2014
10	Terpene	Geosmin	1,779,691	1,799,445	+ +	yes	
11	Bacteriocin	Bacteriocin	1,813,217	1,822,954	+ +	no	Goto et al., 2010; Gaskell et al., 2012
12	PKS (Type I)	Undecylprodigiosin	2,070,646	2,116,351	±	yes	
13	Siderophore	Siderophore	2,199,411	2,209,697	±	yes	Jeong et al., 2016
14	PKS (Type II)	Gray spore pigment	2,673,706	2,742,219	±	yes	
15	Terpene	Epi-isozizaene (Albaflavenone)	2,804,344	2,824,905	+	yes	Takano et al., 2001; Mehra et al., 2008; Jeong et al., 2016
16	PKS (Type II)	Actinorhodin	2,928,724	3,001,218	+ +	yes	
17	NRPS	Calcium-dependent antibiotic (CDA)	4,738,332	4,817,054	±	yes	this study
18	Siderophore	Desferroxamine B	5,290,107	5,301,891	+ + +	yes	Jiang et al., 2006
19	Melanin	Melanin	5,384,385	5,394,954	+	yes	
20	Ectoine	Ectoine	6,336,408	6,346,806	+ + +	yes	this study
21	PKS (Type III)	1,3,6,8-tetrahydroxynaphthalene	7,041,742	7,082,866	+	yes	Hobbs et al., 1990
22	Bacteriocin	Bacteriocin	7,570,510	7,580,725	+	yes	
23	NRPS	Coelichelin	7,826,445	7,877,382	+ +	yes	Jeong et al., 2016
24	Lantipeptide	Class I lantipeptide	8,100,785	8,125,343	+	yes	
25	Terpene	Isorenieratene	8,180,486	8,205,248	+	yes	Davis and Chater, 1990
26	PKS (Type I)	Poly unsaturated fatty acid	8,241,896	8,294,872	+	yes	
27	Terpene	Albaflavenone	8,307,799	8,328,848	±	no	Aaron et al., 2010

*1) Transcription strength measured by TPM values. “-”: < 1; “ ± ”: ≥ 1 - < 10; “ + ”: ≥ 10 - < 100; “ + + ”: ≥ 100 - < 1000; “ + + + ”: ≥ 1000 TPM, cluster with no detected transcription are marked with “n.d.” Described in *Streptomyces coelicolor* A3(2) by Jeong et al. (2016) (”yes” or “no” indicates the described presence or absence in both strains)*

The variety of gene clusters (Table 5) in *S. lividans* TK24 shows that this strain has further potential to be a source of new metabolites of biotechnological relevance.

**FIGURE 8:**
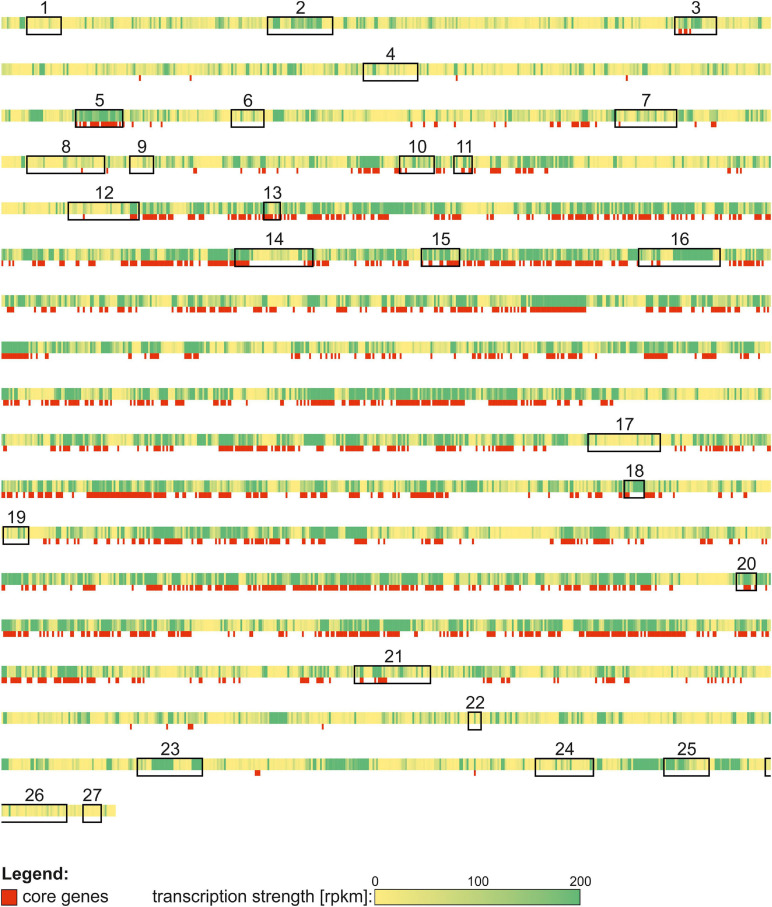
Transcription strength [rpkm] (from yellow to green) and core genes (red) of the *Streptomyces lividans* TK 24 genome. Secondary metabolite gene clusters are marked with boxes and numbered according to Table 5.

In addition, we analyzed whether transcription of genes that belong to the core genome of streptomycetes (present in all of 17 analyzed genomes) might be distinct from that of genes restricted to only certain streptomycetes (non-core), including *S. lividans* TK24. Core genes are predominantly enriched in the middle of the linear genome/chromosome. Transcription of the whole *S. lividans* TK24 genome over the two cultivation conditions and the three time points was used to identify regions of strong or weak transcription (Figure 8). Stronger transcription appears to correlate with the location of core genes closer to the center. Genes located at the ends of the genome rarely belong to the *Streptomycetes* core genome and in most cases are less strongly transcribed.

Among the identified gene cluster, 6 clusters were classified as terpene producing gene cluster. The predicted products are albaflavenone (3x), hopanoids, geosmin and isorenieratene. Albaflavenone was sucessfully produced in *S. coelicolor* A3(2) by Zhao et al. (2008). It functions as an antibiotic for several bacteria by slow down growth. 2-methylisoborneol is a monoterpene which is characterized by its earthy or musty odor. It is produced by several cyanobacteria and actinobacteria and is similar to geosmin. The human nose is very sensitive to these two compunds (Jüttner and Watson, 2007). Hopanoids are triterpenoids which are located in the membrane of several bacteria but also plants and fungi. It was shown that hopanoids are involved in the stability and acid tolerance of the cell membrane. Therefore, it can be assumed that these molecules help cells to adapt to extreme environments (Fischer et al., 2005). Strikingly, hopanoids are not detected in archaea (Belin et al., 2018). The hopanoid producing gene cluster (cluster 5) seems to belong to the core genome of *S. lividans* TK24 in contrast to all other identified secondary metabolite gene cluster (Figure 8). In *Streptomyces scabies*, hopanoid biosynthesis genes are expressed but not essential for growth under laboratory conditions (Seipke and Loria, 2009). Isorenieratene is a light harvesting pigment belonging to the class of carotenoids (Damsté et al., 2001), which was found to be involved in anoxygenic photosynthesis by using hydrogen sulfite as a final electron acceptor instead of oxygen (Brocks et al., 2005). Its biosynthesis was observed in cyanobacteria and a few actinomycetes, like *Streptomyces griseus* (Krügel et al., 1999). The transcriptional regulation was described to be sigB dependent (Lee et al., 2001).

The variety of gene clusters (Table 5) in *S. lividans* TK24 shows, that this strain has further potential to be a source of new metabolites of biotechnological relevance.

In addition, we analyzed whether transcription of genes that belong to the core genome of streptomycetes (present in all of 17 analyzed genomes) might be distinct from that of genes restricted to only certain streptomycetes (non-core), including *S. lividans* TK24. Core genes are predominantly enriched in the middle of the linear genome/chromosome. Transcription of the whole *S. lividans* TK24 genome over the two cultivation conditions and the three time points was used to identify regions of strong or weak transcription (Figure 8). Stronger transcription appears to correlate with the location of core genes in the center of the genome. Genes located at the ends of the genome rarely belong to the *Streptomycetes* core genome and in most cases are less strongly transcribed except some of the secondary metabolite gene clusters, located at the left or right arm of the genome.

**FIGURE 9:**
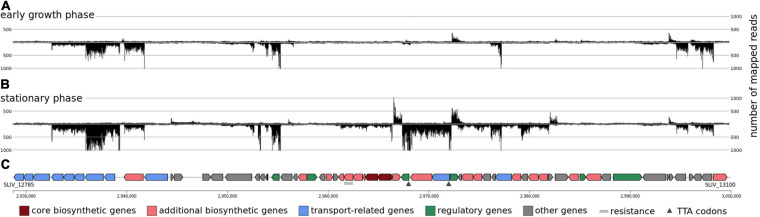
The transcriptional organization of the actinorhodin biosynthetic gene cluster. Screenshot from ReadXplorer 2 (Hilker et al., 2016) depicting gene transcription during early **(A)** and stationary phase **(B)** growth phase. **(C)** denotes the antiSMASH 5.0 (Blin et al., 2019) predictions of the transcribed CDS.

However, two of the secondary metabolite gene clusters seem to be less strongly transcribed under the analyzed conditions, although they are located in the center of the chromosome (e.g., clusters 17 and 19). These two clusters do not contain any core genes and the activation of these clusters is not growth phase dependent. However, cluster 16, encoding genes of the actinorhodin biosynthesis, was strongly transcribed in the stationary phase but does not belong to the streptomycetes core genome. It is a PKS type II gene cluster which consists of 64 genes (*SLIV_12785*to *SLIV_13100*). The transcriptional landscape of the actinorhodin gene cluster was compared between different cultivation time points in minimal media containing casamino acids (Figure 9). It is interesting to note that actinorhodin is produced at the onset of the stationary phase of growth. This correlates well with the transcriptional profiles (Figure 9). During normal growth, only few genes of the cluster are transcribed, all of them known as regulatory genes. During transition to stationary phase, however, the whole cluster is strongly transcribed. Actinorhodin is an aromatic polyketide produced by *S. coelicolor* and *S. lividans* strains (Magnolo et al., 1991). In *S. coelicolor* A3(2) it is produced only in the stationary phase (Gramajo et al., 1993). Its biosynthesis is oxygen-dependent (Magnolo et al., 1991) and improved through iron limitation (Coisne et al., 1999). These findings are consistent with the transcription of the actinorhodin biosynthesis genes in *S. lividans* TK24 observed here (Figure 9).

## Methods

### Cultivation Conditions of *S. lividans* TK24

*Streptomyces lividans* TK24 cultivation was carried out in bioreactor cultivations. Bioreactors were inoculated with biomass harvested from a two-step preculture in phage medium (10 g L^1^ glucose, 5 g L^1^ tryptone, 5 g L^1^ yeast extract, 5 g L^1^ Lab Lemco powder, 0.74 g L^1^ CaCl2 × 2H_2_O, 0.5 g L^1^ MgSO_4_ x 7H_2_O, pH: 7.2). A loop of frozen mycelium was transferred to 100 mL phage medium and incubated at 30°C for 72 h under moderate stirring. Next, 25 mL culture was centrifuged, supernatants removed, and the pellet was resuspended in 100 mL fresh medium and incubated for 24 h under the same conditions. This culture was harvested, centrifuged, and washed 3 times in fresh bioreactor medium. A ratio of 25 mL preculture per liter bioreactor volume throughout all experiments was maintained resulting in an average start cell dry weight of 20 mg L^1^. Experiments for transcriptomics were performed in a BioFlo3000 bioreactor (Eppendorf) filled with 3.5 L medium. Experiments for proteomic analysis were performed in a DASGIP parallel bioreactor system (Eppendorf) filled with 1 L medium. Temperature, agitation, aeration, and pH were set at 30°C, 400 rpm (BioFlo3000)/500 rpm (DASGIP), 120 standard L h^1^ (0.4% vvm) (BioFlo3000)/60 standard L h^1^ (1% vvm) (DASGIP) and 6.8, respectively. pH control was done by the addition of 1M KOH or 1M H_2_SO_4_ into BioFlo3000 vessels and 4M KOH or 2M H_2_SO_4_ to DASGIP vessels. 1 mL antifoam Y-30 emulsion (Sigma-Aldrich) was added to avoid formation foam. Bioreactor experiments are performed in minimal medium (10 g L^1^ glucose, 3 g L^1^ (NH_4_)_2_SO_4_, 2.6 g L^1^ K_2_HPO_4_, 1.8 g L^1^ NaH_2_PO_4_, 0.6 g L^1^ MgSO_4_ x 7H_2_O, 1 mg L^1^ ZnSO_4_ x 7H_2_O, 1 mg L^1^ FeSO_4_ x 7H_2_O, 1 mg L^1^ CaCl_2_, 1 mg L^1^ MnCl_2_ x 4H_2_O) and minimal medium supplemented with casamino acids (5 g.L-1 Casamino Acids Technical). Mid-log, late-lag and stationary phase samples were taken. For transcriptomic analysis, 8 × 1 mL samples per time point were centrifuged at 21,000 × *g* for 30 s, supernatant was removed, and pellets were snapfrozen in liquid nitrogen. For proteomic analysis, samples of 10 mL were centrifuged at 3220 × *g* for 10 min, supernatant was transferred and filtrated over 0.2 PES membrane, pellet and supernatant were snapfrozen in liquid nitrogen. All samples were stored at −80°C upon further analysis.

### Total RNA Isolation and Sequencing of cDNA Libraries Made From mRNA

RNA was isolated using a Qiagen RNeasy mini kit in combination with an RNase-free DNase kit (Qiagen, Hilden, Germany). Absence of DNA was assayed by PCR with primers binding to genomic *S. lividans* TK24 DNA. RNA quantity as well as quality were checked with the Trinean Xpose system (Gentbrugge, Belgium) and an Agilent RNA 6000 Pico kit run on an Agilent Bioanalyzer 2100 (Agilent Technologies, Böblingen, Germany). RNA was isolated from *S. lividans* TK24 fermenter cultures grown in minimal media with and without casamino acids. Of each cultivation condition, RNA was isolated from early and late growth phase as well as from stationary phase.

Two different cDNA libraries were prepared from the RNA samples. The RNAs for the sequencing of 5′-ends of primary transcripts were pooled prior to library construction whereas the RNAs for whole transcriptome libraries were handled separately. Prior to library construction stable RNA was depleted by using the Ribo-Zero kit (Epicentre, Madison, WI, United States). The manufacturer’s instructions were adjusted due to the high G + C content of *S. lividans* TK24 and the potentially resulting secondary structures of the transcripts. Therefore, the incubation temperature of the probe hybridization samples with magnetic beads was elevated from 50°C to 65°C. Successful rRNA depletion was checked by an Agilent RNA 6000 Pico kit run on an Agilent Bioanalyzer 2100 (Agilent Technologies, Böblingen, Germany). The protocol for the 5′-end library was conducted as described previously (Pfeifer-Sancar et al., 2013) with the modifications according to (Irla et al., 2015). The resulting 5′-enriched cDNA libraries were sequenced on the MiSeq system (Illumina, San Diego, CA, United States). The whole transcriptome library was prepared essentially according to the standard protocol of the TruSeq Stranded mRNA Library Prep Kit (Illumina, San Diego, CA, United States) but omitting the polyA-purification step. Both cDNA libraries were sequenced using TruSeq kits (Illumina, San Diego, CA, United States). The 5′-enriched library was sequenced on a MiSeq system (Illumina, San Diego, CA, United States) in single read (75 nt) mode. The whole transcriptome library was sequenced on a HiSeq 1500 sequencer (Illumina, San Diego, CA, United States) in paired end mode (2 × 75 nt).

### Read Mapping and Determination of Transcription Start Sites

After Illumina base-calling and demultiplexing with Illumina bcl2fastq2 Conversion Software v2.19.1trimming was performed with the software Trimmomatic v0.33 (Bolger et al., 2014) and mapping with Bowtie v2.2.7 (Langmead and Salzberg, 2012) with standard parameters except for increased pair size (-X 600) for the whole transcriptome data set. For the 5′-end enriched library reads were trimmed to 25 bp and for the whole transcriptome library paired-end reads were quality trimmed only. Both were mapped to the already published genome sequence of *S. lividans* TK24 (Rückert et al., 2015) with a minimum read length of 20 nt.

For examination and visualization of the RNA-seq data, the software ReadXplorer 2 (Hilker et al., 2016) was used. The tools included in ReadXplorer 2 were used for TSS and operon prediction as well as for the identification of novel transcripts. The automatic classification of intragenic TSS was used to examine potential changes of incorrectly predicted translation start sites. Initially, putative TSS were automatically predicted with a minimum of 10 read starts and a coverage increase from −1 to + 1 of 1000%. The maximal distance of the putative TSS to the next TLS was set to 500 nt. The described settings were empirically established with a random set of TSS and resulted in good signal to noise ratio as well as specificity (data not shown). To detect low abundance transcripts and their corresponding TSS the low coverage setting of min. 10 read starts and a coverage increase of 100% was chosen. All predicted TSS were manually reviewed to exclude false positive results. TSS with inconclusive or indistinct read stacks were discarded, which often applies to highly transcribed regions downstream of a primary TSS. Coding sequences were automatically predicted as transcribed polycistronically, if at least five reads of the whole transcriptome data set bridged the intergenic regions of the genes.

Putative translation start site (TLS) alterations were called automatically by ReadXplorer 2 and the described TSS prediction tools, if an intragenic TSS, as well as an in-frame start codon (ATG, GTG, TTG) downstream of this TSS, was identified. For this, the first 25% of the nucleotides of every CDS were analyzed. The TLS was only altered, if read starts were also manually identified in the whole transcriptome track and no reads in this track were mapped to the originally predicted TLS. A further condition was that no additional TSS upstream of the intragenic TSS was identified. As a further control step, the conservation of the TLS for the corresponding CDS was verified by BLASTX (Altschul et al., 1997) using standard NCBI parameters.

### Proteomics Experimental Design and Statistical Rationale

For the proteomic characterization of *S. lividans* TK24 secretome 6 to 8 biological repeats were prepared for each experimental condition. Raw MS files from the mass spectrometer were analyzed by MaxQuant v1.5.3.30, a quantitative proteomics software package designed for analyzing large mass spectrometric data sets (Cox and Mann, 2008). MS/MS spectra were searched against the re-annotated *S. lividans TK24* proteome (Rückert et al., 2015) (7505 proteins) and common contaminants, using the Andromeda search engine (Cox et al., 2011). Enzyme specificity was set to trypsin, allowing for a maximum of two missed cleavages. Dynamic (methionine oxidation and N-terminal acetylation) and fixed (S-Carbamidomethylation of cysteinyl residues) modifications were selected. Precursor ion mass tolerance was set to 20 ppm and fragment ion tolerance to 20 ppm for Orbitrap QE or 0.5 Da for Orbitrap Elite. Protein and peptide False Discovery Rate (FDR) were set to 1%. Peptide features were aligned between different runs and masses were matched (“match between runs” feature), with a match time window of 2 min and a mass alignment window of 20 min. Label-free, relative protein quantification was performed using the iBAQ algorithm through the MaxQuant software (Cox and Mann, 2008; Schwanhäusser et al., 2011).

Data analysis (filtering, transformation, and statistical analysis) was performed using custom scripts in R language (R Core Team, 2017). Functional characterization of the detected proteins was performed based on the manually annotated proteome of *S. lividans* obtained from SToPSdb^2^ (Tsolis et al., 2018).

### LC-MS/MS Analysis

Lyophilized peptide samples were first dissolved in an aqueous solution containing 0.1% v/v formic acid (FA) and 5% v/v ACN and were analyzed using nano-Reverse Phase LC coupled to a Q Exactive^TM^ Hybrid Quadrupole - Orbitrap or Orbitrap Elite Hybrid Iontrap - Orbitrap mass spectrometer (Thermo Scientific, Bremen, Germany) through a nanoelectrospray ion source (Thermo Scientific, Bremen, Germany). Peptides were initially separated using a Dionex UltiMate 3000 UHPLC or a Thermo EASY-nLC^TM^ -1200 system on an EasySpray C18 column (Thermo Scientific, OD 360 μm, ID 50 μm, 15 cm length, C18 resin, 2 μm bead size) at a nanoLC flow rate of 300 nL min^–1^. The LC mobile phase consisted of two different buffer solutions, an aqueous solution containing 0.1% v/v FA (Buffer A) and an aqueous solution containing 0.1% v/v FA and 80% v/v ACN (Buffer B). A 60 min gradient was used from Buffer A to Buffer B (percentages from each in parentheses below) as follows: 0–3 min constant (96:4), 3–35 min (65:35); 35–40 min (35:65); 40–41 min (5:95); 41–50 min (5:95); 50–51 min (95:5); 51–60 min (95:5). Peptides were analyzed in the Orbitrap QE or an Orbitrap Elite as separate complete experimental datasets. Orbitrap QE operated in positive ion mode (nanospray voltage 1.6 kV, source temperature 250°C), in data-dependent acquisition (DDA) mode with a survey MS scan at a resolution of 70,000 FWHM for the mass range of 400-1,600 *m/z* for precursor ions, followed by MS/MS scans of the top 10 most intense peaks with + 2, + 3, and + 4 charged ions above a threshold ion count of 16,000 at a resolution of 35,000 FWHM. Orbitrap Elite was operated in positive ion mode (nanospray voltage 1.8 kV, source temperature 275°C), in DDA mode with a survey scan at a resolution of 240,000 FWHM for a mass range of 375-1500 *m/z* for precursor ions, followed by MS/MS scans of the 20 most intense peaks with charge + 2 or higher, above a threshold count of 500 at a resolution of 17,000 FWHM. MS/MS in Orbitrap QE was performed using normalized collision energy (NCE) of 25% with an isolation window of 3.0 *m/z*, an apex trigger 5–15 s and a dynamic exclusion of 10 s. In Orbitrap Elite, MS/MS collisional induced dissociation (CID) was performed using 35% NCE with an isolation window of 2.0 *m/z*, and a dynamic exclusion list of 30 s. Data were acquired with Xcalibur 2.2 software (Thermo Scientific).

### Additional Software Tools

#### Re-Annotation

As a basis, the original annotation (Rückert et al., 2015) done with the prokka pipeline (Seemann, 2014) was used and compared to the automated annotation provided by RefSeq as well as to the *S. coelicolor* A3(2) genome (AL645882), using EDGAR (Blom et al., 2009). Additional CDS and differences in the translation start sites were manually compared and resolved in GenDB (Meyer et al., 2003) using the transcriptome and proteome data obtained in this study (see above). For tRNA detection the tool tRNAscan-SE 1.21 (Schattner et al., 2005) and ARAGORN (Laslett and Canback, 2004) as part of the prokka pipeline (Seemann, 2014) were used with standard parameters. Possible protein coding regions in novel transcripts were examined through BLASTX (Altschul et al., 1997). ARNold (Naville et al., 2011) was applied to find transcriptional terminators. The software package Infernal (Nawrocki and Eddy, 2013) with Rfam as a database (Nawrocki et al., 2015) was used with an E-value cut-off of 0.01 and standard parameters otherwise.

antiSMASH 5.0 (Blin et al., 2019) was used for the prediction of biosynthetic gene clusters. Promoter motif prediction was carried out using the tool Improbizer (Ao et al., 2004). Therefore, 80 nt upstream of all annotated TSS were used as input for Improbizer. For the prediction of the core genome, EGDAR (Blom et al., 2009) was used with the following *Streptomyces* genomes besides that of *S. lividans* TK24: *S. coelicolor* A3(2) (AL645882), *S. avermitilis* MA-4680 (BA000030), *S. cattleya* DSM 46488 (CP003219), *S. collinus* Tu 365 (CP006259), *S. davawensis* JCM 4913 (HE971709), *S. pratensis* ATCC 33331 (CP002475), *S. fulvissimus* DSM 40593 (CP005080), *S. griseus* subsp. griseus NBRC 13350 (AP009493), *S. hygroscopicus* subsp. jinggangensis 5008 (CP003275), *S. rimosus* subsp. rimosus ATCC 10970 (ANSJ00000000), *S. scabiei* 87.22 (FN554889), *Streptomyces* sp. PAMC26508 (CP003990), *Streptomyces* sp. SirexAA-E (CP002993), *S. venezuelae* ATCC 10712 (FR845719), *S. violaceusniger* Tu 4113 (CP002994), and *S. albidoflavus* (CP004370).

## Conclusion

In this study, the genome annotation of *S. lividans* TK24 was noticeably improved by the use of transcriptome and proteome data. The detailed analyses presented here provide a basis for future studies to the scientific community working on Streptomyces and highlight the importance of a high quality genome and up to date annotation. An accurate genome annotation is the basis for subsequent research regarding biochemical pathways or biotechnological optimization and use of this strain. It is needed for genetic engineering as well as omics-based experiments.

The identification of transcription start sites, promoter elements and the analysis of the operon structure is very important for a better understanding of transcriptional regulation in *S. lividans* TK24 as a model organism for several *Streptomyces* spp. Several regulatory elements, like riboswitches or antisense RNAs were described, which could help to understand the regulation of single pathways.

The identification of secondary metabolite gene clusters and the transcriptional organization of these clusters play an important role for the identification of novel biotechnological products. Furthermore, the up to date identification of gene clusters and genomic regions, which seem to be not transcribed, could point out targets for genome reduction and therefore decrease metabolic burden during the production of industrial relevant products.

## Data Availability Statement

The datasets presented in this study can be found in online repositories. All RNAseq data is available via the SRA project SRP144344. The updated genome is accessible via GenBank accession CP009124. All proteomic data is available via PRIDE projects PXD009675, PXD006819, and PXD006818.

## Author Contributions

AE designed, planned, and interpreted the experimental work of this study and supervised the proteomics work. KB carried out bacterial fermentation. TB carried out the transcriptomic experiments. KS and MH carried out analysis of the proteomic experiments. JD carried out all analyses of the transcriptomic data and drafted the manuscript. JA, JK, TB, AE, and CR assisted in interpreting the data and revised the manuscript. JK and TB coordinated this study. All authors read and approved the final manuscript.

## Conflict of Interest

The authors declare that the research was conducted in the absence of any commercial or financial relationships that could be construed as a potential conflict of interest.

## Footnotes

1 http://stopsdb.eu

2 http://www.stopsdb.eu

## Supplementary Material

Supplementary Table 1 Novel coding regions.

Supplementary Table 2 Corrections of translation start sites.

Supplementary Table 3 Protein-coding genes validated by proteome data.

Supplementary Table 4 Transcription start sites (TSS).

Supplementary Table 5 Promoter analysis.
